# Development of P301S tau seeded organotypic hippocampal slice cultures to study potential therapeutics

**DOI:** 10.1038/s41598-021-89230-3

**Published:** 2021-05-13

**Authors:** James M. McCarthy, Jasmeet Virdee, Jessica Brown, Daniel Ursu, Zeshan Ahmed, Annalisa Cavallini, Hugh N. Nuthall

**Affiliations:** 1 Neuroscience, Eli Lilly and Company, Erl Wood Manor, Windlesham, Surrey GU20 6PH UK; 2 Present Address: Astex Pharmaceuticals, Milton Road, Cambridge, CB4 0QA UK; 3 Present Address: Boehringer Ingelheim, Birkendorfer Straβe 65, Biberach, Germany

**Keywords:** Drug screening, Target validation, Drug discovery, Mechanisms of disease, Cellular neuroscience, Diseases of the nervous system

## Abstract

Intracellular tau inclusions are a pathological hallmark of Alzheimer's disease, progressive supranuclear palsy, corticobasal degeneration and other sporadic neurodegenerative tauopathies. Recent in vitro and in vivo studies have shown that tau aggregates may spread to neighbouring cells and functionally connected brain regions, where they can seed further tau aggregation. This process is referred to as tau propagation. Here we describe an ex vivo system using organotypic hippocampal slice cultures (OHCs) which recapitulates aspects of this phenomenon. OHCs are explants of hippocampal tissue which may be maintained in culture for months. They maintain their synaptic connections and multicellular 3D architecture whilst also permitting direct control of the environment and direct access for various analysis types. We inoculated OHCs prepared from P301S mouse pups with brain homogenate from terminally ill P301S mice and then examined the slices for viability and the production and localization of insoluble phosphorylated tau. We show that following seeding, phosphorylated insoluble tau accumulate in a time and concentration dependent manner within OHCs. Furthermore, we show the ability of the conformation dependent anti-tau antibody, MC1, to compromise tau accrual in OHCs, thus showcasing the potential of this therapeutic approach and the utility of OHCs as an ex vivo model system for assessing such therapeutics.

## Introduction

Soluble microtubule-associated protein tau assembles into insoluble, filamentous, and hyperphosphorylated intracellular inclusions in several human neurodegenerative diseases, collectively known as tauopathies. These include Alzheimer's disease (AD), progressive supranuclear palsy (PSP), corticobasal degeneration (CBD), Pick's disease, Lytico-Bodig disease, chronic traumatic encephalopathy and frontotemporal dementia with parkinsonism linked to chromosome 17 (FTDP-17). More than 50 pathogenic mutations have been identified in the MAPT gene, some of which have been linked directly to early onset familial forms of dementia (such as FTDP-17), establishing that tau alterations alone can cause neurodegeneration^1–3^.

Tau pathology appears to spread in a progressive and stereotypical fashion. For example, in AD, misfolded, hyperphosphorylated tau first accumulates in the locus coeruleus, from where it appears to spread to the entorhinal cortex, hippocampus, and neocortex. This differential distribution underlies the Braak stages of tau pathology^4,5^. Recently, this propagation effect has been demonstrated experimentally in a number of models, both animal^6–8^ and cellular^9–16^.

Organotypic hippocampal slice cultures (OHCs) are a particularly useful model to study the accumulation of aggregated tau as they combine some of the finest features of the in vivo and cellular models—they are a 3D multicellular system in which synaptic connections are largely maintained and offer direct access for various imaging methods, biochemical assays and electrophysiology techniques. For this reason, there is now a number of studies where they have been employed to study various aspects of neurodegenerative diseases^17–21^. OHCs generated from transgenic mice expressing mutant human tau may produce aggregated and phosphorylated tau. For example, cortical-hippocampal slices generated from JNPL3 mice produce insoluble tau after 19 days in vitro (DIV)^22^. Hippocampal slices produced from pro-aggregant tau_RD_KΔ280 mice produce insoluble phosphorylated tau and ThioS positive tau aggregates at 25 DIV^23^. These slices demonstrate mislocalization of tau to somata and apical dendrites, reduced dendritic spine density, reduced intracellular calcium following KCl stimulation, increased caspase 3 activation and neuronal death. Hippocampal slices from P301S mice^24^ produce phosphorylated tau aggregates 10 days after seeding with synthetic preformed fibrils^25^. Seeded slices displayed impaired amplitude and Long-Term Potentiation of population spikes in the CA1, versus mock treated controls. Thus, OHCs offer a very appealing model of tauopathies as they recapitulate many of the features of pathogenesis seen in vivo, in a fully accessible in vitro environment.

To date few, if any, studies have fully characterized the accumulation and distribution of insoluble phosphorylated tau over time, nor the effect of seeding slices with tau aggregates from diseased mice. Rather, the slices have been used to measure the level of insoluble or aggregated tau at a single time point, often following a treatment. In the present study, we sought to rectify this by examining the time course, kinetics, localization and effect of insoluble phosphorylated tau accumulation in seeded organotypic hippocampal slices from human P301S tau transgenic mice. Having established and fully profiled this seeding model of tau accumulation, we then demonstrated the ability of a conformation dependent anti-tau antibody to inhibit this process, in an antibody concentration dependent manner.

## Results

### Organotypic hippocampal slice cultures maintain their architecture and comprise all the major cell types of the brain parenchyma after 2 weeks in culture

**Figure 1:**
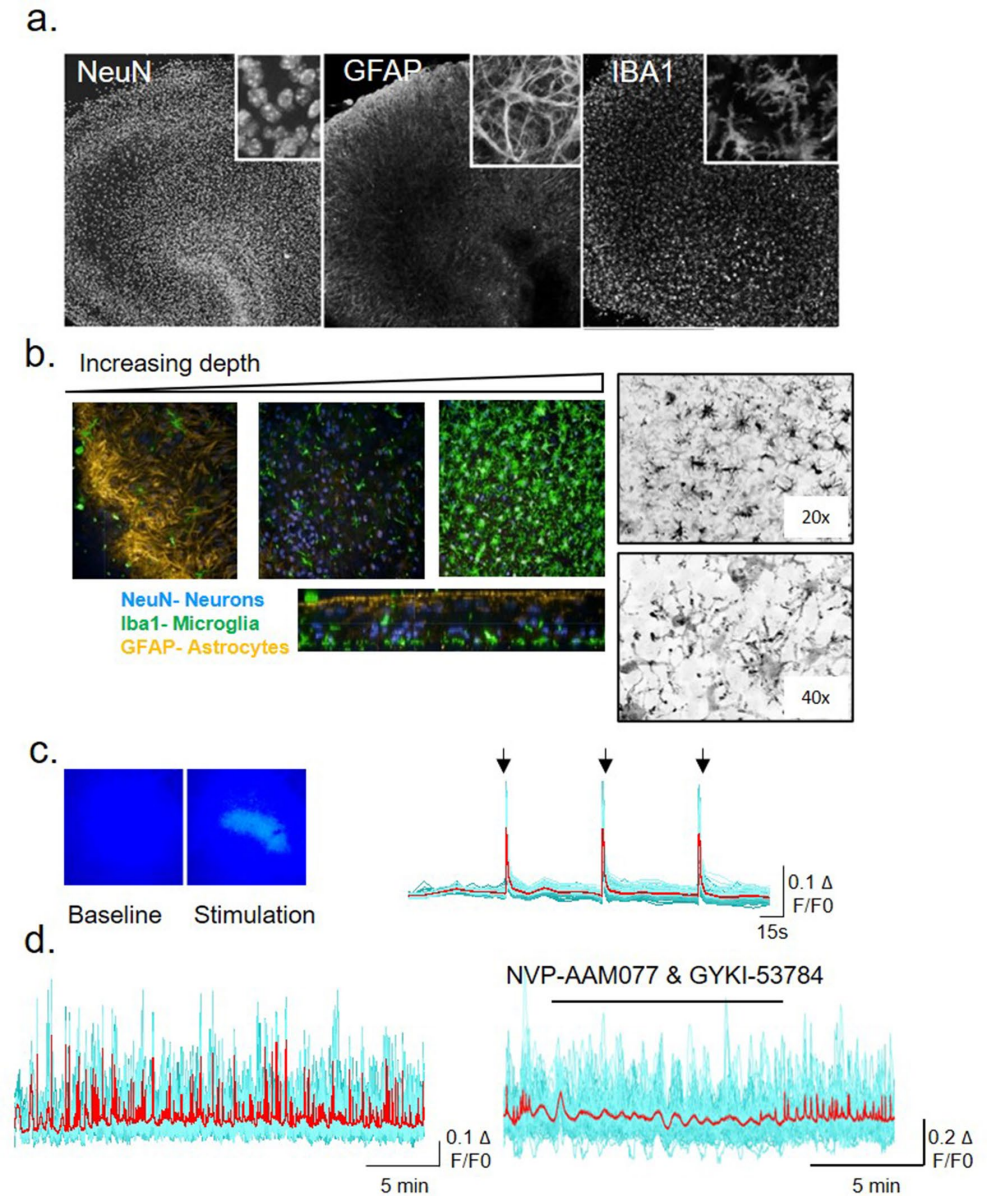
P301S OHCs maintained their architecture and comprised multiple functional cell types, possessing homeostatic microglia after 2 weeks in culture. (**a**) OHCs maintained their hippocampal structure over time in culture and expressed multiple cell types: neurones, astrocytes and microglia—labelled with NeuN, GFAP and IBA1 respectively. (**b**) Beyond 2 weeks in culture, a glial scar was observed over the top of the OHCs, with neurones being dispersed throughout the culture, and microglia migrating to the bottom towards the membrane. P2RY12 staining revealed homeostatic microglia are present throughout the slice. (**c**) Electrical field stimulation of OHCs (10 V, 10 s AT 25 Hz—black arrows) showed there was no change in the amplitude of the calcium response with repeated stimulation. N = 7. (**d**) Calcium imaging of OHCs demonstrated that 3/7 slices were spontaneously active—with multiple calcium oscillations that could be blocked by GYKI-53784 and NVP-AAM07 (AMPA and NMDA antagonist, respectively).

To characterise the cellular composition and architecture of the 3D OHCs, slices were fixed and stained at 2 weeks in vitro for multiple cell lineage markers. We observed the preservation of the classic neuronal architecture of the hippocampus, e.g. dentate gyrus and CA1 region, throughout the culture period (Fig. 1a). There was a heterogeneous display of cell types throughout the OHC section, with astrocytes forming a glial scar at the surface of the culture. Microglia were dense and ramified at the bottom of the culture, with neurones being distributed evenly throughout the slice. Homeostatic, highly branched microglia were observed after 14 days in vitro as shown with P2RY12 staining (Fig. 1b). This observation indicates that over time in culture microglia assume a resting phenotype, following trauma of the invasive slicing procedure.

### Calcium imaging confirm that OHCs are functionally active

A functional assessment of neuronal activity in the OHCs was carried out by calcium imaging. Slices were incubated with Fluo4-AM in the presence of 1% pluronic acid for 1 h, and subsequently recorded for calcium dynamics and changes in fluorescence over time. A proportion of slices (43%) displayed spontaneous activity, which was subsequently blocked with a selective non-competitive AMPA receptor antagonist, GYKI-53784, and a NMDA receptor antagonist, NVP-AAM007 (Fig. 1d). The changes in intracellular calcium were again observed on wash out of the compounds. We also repeatedly electrically stimulated the OHCs (10 V, 10 stimuli) and were able to record the resultant changes in fluorescence (10 frames/second). A stable peak calcium response could be observed upon repeated stimulation (Fig. 1c). This data indicated that slices are functionally active and viable at 2 weeks in culture.

### Seeding with P301S brain homogenate and nature of tau produced

Once we had established that OHCs maintained both their functionality and architecture throughout their time in culture, we proceeded to use slices prepared from the P301S mouse model to study tauopathy within the hippocampus.

P301S tau transgenic mice overexpress the 0N4R isoform of human tau containing the P301S mutation under the murine *thy1* promoter^26^. Homozygous P301S mice develop intracellular tau inclusions (hyperphosphorylated and fibrillar), severe paraparesis, and extensive neurodegeneration by 5–6 months of age. The pathology is most severe in the brainstem and spinal cord with minor involvement of the forebrain^26^.

We first assessed the production of insoluble phosphorylated tau in ex vivo hippocampal tissue culture slices cultured for 0 to 8 weeks via immunoblotting. For comparison, hippocampi from live Tg P301S mice of various ages were harvested and examined in tandem (Supp Fig. 1a,b). Insoluble phosphorylated tau was first detectable in the hippocampi of adult P301S mice via fractionation and immunoblot at ~ 16 weeks (not cultured; in vivo control for comparison). In OHCs no insoluble phosphorylated tau was detectable after up to 8 weeks in culture. In both cases the level of total tau remained constant (Supp Fig. 1b). The levels of human tau observed with CP27 staining over time showed uniform expression throughout the hippocampus, particularly within CA1 and CA3 region in different segments of the hippocampus (Supp Fig. 1c).

**Figure 2:**
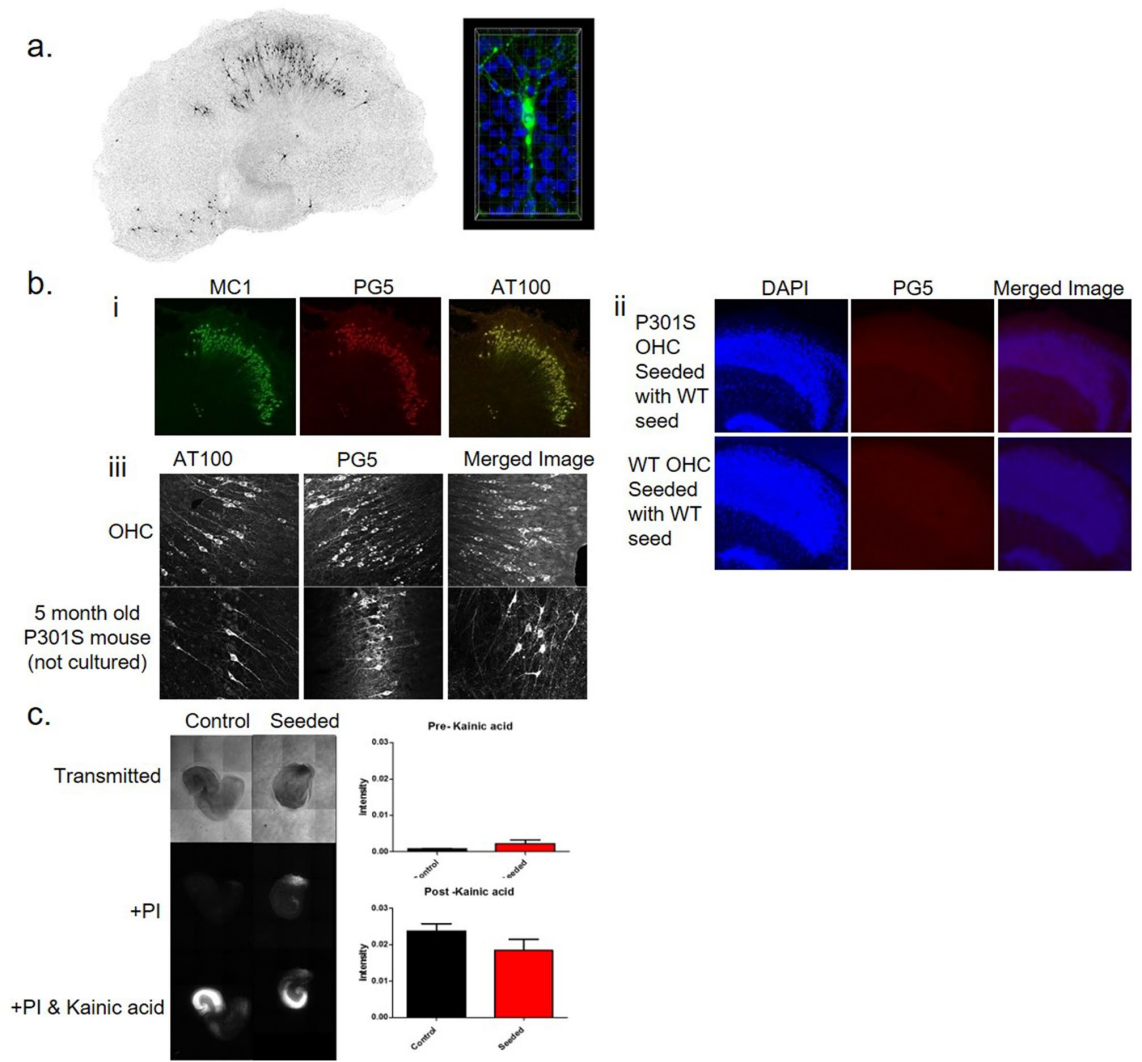
Inoculating P301S slices with P301S brain homogenate induced hyperphosphorylated tau inclusions that had no significant effect on the toxicity of the OHCs. (**a**) Inoculation of OHCs induced AT100 positive tau inclusions that were observed throughout the CA1 region of the hippocampus. Single neurones displayed large perinuclear inclusions and these were visible throughout the neurites. (**bi**) Tau inclusions are positively labelled for PG5, MC1 and AT100  (**bii**) WT slices, or P301S slices seeded with WT seed, do not show these positive tau inclusions. (**biii**) Similar tau staining (AT100 and PG5) was observed between OHCs and non-cultured slices from terminally ill P301S mice at 5 months (**c**) Inoculation of P301S seed to the OHCs at DIV 0 and subsequent culturing for 4 weeks showed no significant increase in neuronal death as shown with PI staining. Incubation of 20 µM Kainic acid induced neuronal death and subsequently an increase in PI fluorescence confirming that the slices were viable and contained live neurones.

Recent in vitro and in vivo studies have shown that tau aggregates may seed further tau aggregation within neurons and spread to neighbouring cells and functionally connected brain regions^6,7,9^. To determine if seeding of OHCs could accelerate or indeed enable the production of insoluble phosphorylated tau, we inoculated slices with brain homogenate from terminally ill P301S mice (5 nM) and fixed and stained for various markers of conformationally altered tau after 14 day in vitro (Fig. 2a). AT100 is believed to recognize human PHF tau and tau phosphorylated at the pathological epitopes T212/S214 (AT100); PG5 recognizes tau phosphorylated at at the pathological epitopes S409; MC1 is a conformation dependent antibody which recognizes neurofibrillary tangles. AT100, PG5 and MC1 positive tau was present in the CA1 region of the hippocampus and to a lesser extent within the CA3 in all seeded OHCs imaged (Fig. 2bi), but not in WT or P301S OHCs seeded with WT seed (Fig. 2bii). Within single hippocampal neurones we also stained for and observed large perinuclear inclusions of AT100 positive tau.

For comparison, 100 µM hippocampal slices were also produced from terminally ill P301S mice and processed in an identical manner (not cultured; proceeded direct to staining to have a comparison to in vivo tau aggregates). Both the slices from terminally ill mice and the OHCs displayed tau inclusions which were positive for MC1, PG5 and AT100, indicating that the nature of the tau being produced ex vivo in the OHCs is comparable to what occurs in vivo. Additionally, the same slices were positive for both AT100 and PG5 indicating that both phosphorylated and hyperphosphorylated tau are present (Fig. 2biii).

### Toxicity effect of seeding

To determine a potential cytotoxic effect of seeding, the P301S OHCs were stained with propidium iodide (PI), a fluorescent stain which is not permeant to live cells but diffuses rapidly in cells with compromised membrane integrity. Slices were cultured for up to 4 weeks with or without the addition of P301S seed before being assessed for viability (Fig. 2c). Cultures treated with seed showed no gross increase in cell death compared to control slices assessed via PI uptake. In control and seeded slices, the addition of kainic acid confirmed that the slices were viable and contained live cells (Fig. 2c).

###  Concentration response of OHCs inoculation

**Figure 3:**
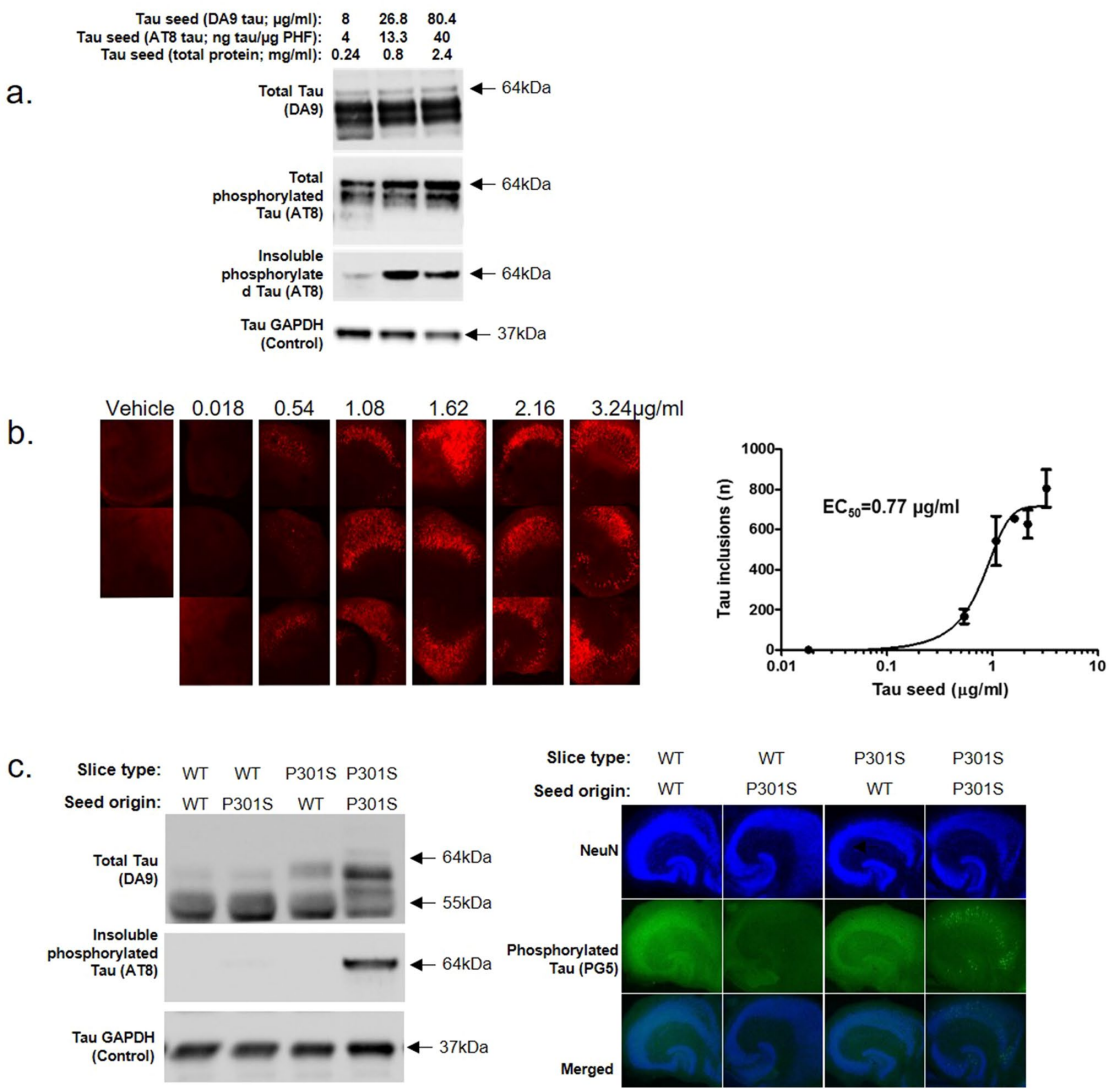
A robust and concentration dependent accumulation of insoluble phosphorylated tau in P301S OHCs seeded with P301S seed. (**a**) OHCs from P7 P301S mice were seeded immediately after preparation with various concentrations of brain homogenate from terminally ill P301S mice or aged matched WT mice. Slices were cultured for 2 weeks, lysed, fractionated and analyzed via immunoblot. Soluble or S1 fraction: Total tau (DA9), Total phosphorylated tau (AT8), GAPDH (Control). Insoluble pellet: phosphorylated tau (AT8). The maximum production of insoluble tau was seen with a seed comprising of 13.3 ng tau/µg PHF. Western blot image cropped for focus. For full original image see Supp Fig. 2. (**b**) These data could also be replicated using immunolabelling, where there was a visible increase in the number of MC1 positive tau inclusions with an increase in application of brain homogenate containing seed, an EC_50_ value of 0.77 µg/ml could be obtained. (**c**) OHCs from P7 P301S and WT mice cultured for 1 week were, lysed, fractionated and analyzed via immunoblotting for insoluble phosphorylated tau or fixed and analyzed for PG5 + tau via IHC. Only P301S slices seeded with P301S brain homogenate produced a detectable level of insoluble and phosphorylated tau. Western blot image cropped for focus. For full original image see Supp Fig. 2.

The most efficient concentration of seed for inducing the production of insoluble phosphorylated tau in OHCs was determined by inoculating the slices with a range of P301S brain homogenate concentrations, and subsequently analyzing the production of insoluble tau via immunoblotting or via immunolabelling for phosphorylated tau (MC1; Fig. 3a,b). A clear dose response was observed with maximum insoluble tau production achieved with seed consisting of 3.24 µg/ml giving an EC_50_ value of 0.77 µg/ml. Thus, an increase in P301S seed led to an increase in visible MC1 tau inclusions. We also sought to determine whether wildtype (WT) slices could be inoculated with P301S seed and show tau pathology. We observed that WT slices seeded with WT brain homogenate or P301S brain homogenate were unable to induce pathology. The same applied to P301S slices seeded with WT brain homogenate—they did not produce any detectable levels of insoluble phosphorylated tau as determined by both immunoblotting and immunohistochemistry (IHC; Fig. 3c). Only OHCs from P301S mice, seeded with homogenate from terminally ill P301S mice produced insoluble phosphorylated tau after 7 days in culture.

### Time course of insoluble tau production

**Figure 4:**
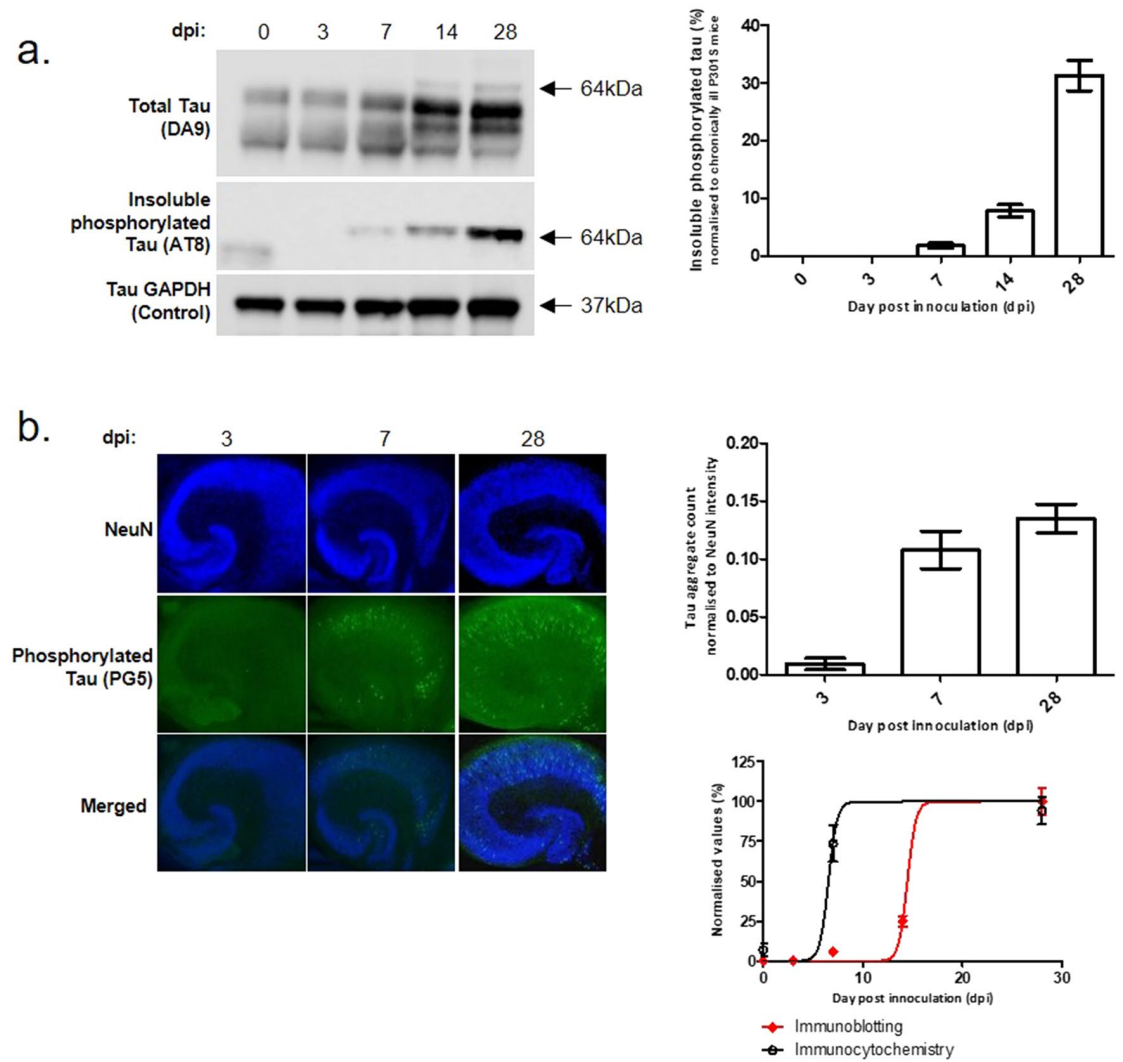
A slow progressive increase in insoluble phosphorylated tau is observed over time in culture. OHCs from P7 P301S and WT mice were seeded immediately after preparation with brain homogenate from terminally ill P301S mice or aged matched WT mice. (**a**) Slices were cultured for 0, 3, 7 or 28 days, lysed, fractionated and analyzed via immunoblotting for insoluble phosphorylated tau or (**b**) fixed and stained for analysis of PG5 + tau. The level of insoluble tau increased over time up to 28 days. Western blot image cropped for focus. For full original image see Supp Fig. 3.

To study the time course of the production of insoluble phosphorylated tau by OHCs following seeding, slices were analyzed 0, 3, 7, 14 or 28 days after inoculation via immunoblotting (Fig. 4a) and IHC (Fig. 4b). Insoluble phosphorylated tau was first detectable at 7 days. The quantity of insoluble tau increased steadily from 7 to 28 days indicating its continuous production. Interestingly, there was a marked difference between the kinetics of phosphorylated tau production detected via immunoblotting and IHC. In both cases, no PG5 + /insoluble tau is detectable at 3 days, whilst the maximum level is observed at 28 days. However, the IHC appears to show more aggressive accumulation of tau than the immunoblotting. This may be due to a technical issue—for example in the immunoblots AT8 primary antibody was used to detect insoluble phosphorylated tau as it gives a stronger signal than PG5. Conversely, in IHC, PG5 was employed as it was found to produce substantially less background than AT8. Alternatively, the difference could be genuine. The early puncta observed via IHC in the OHCs may not be immediately insoluble and so are not detected via fractionation and immunoblotting.

### Antibody neutralization of P301S seed by MC1

Having established a robust and multicellular 3D model of tau seeding and pathology, we wished to determine if we could stop or hinder this process pharmacologically, and thus determine the potential of our OHC model as a screening system for antibodies with the potential to neutralize tau seeds.

**Figure 5:**
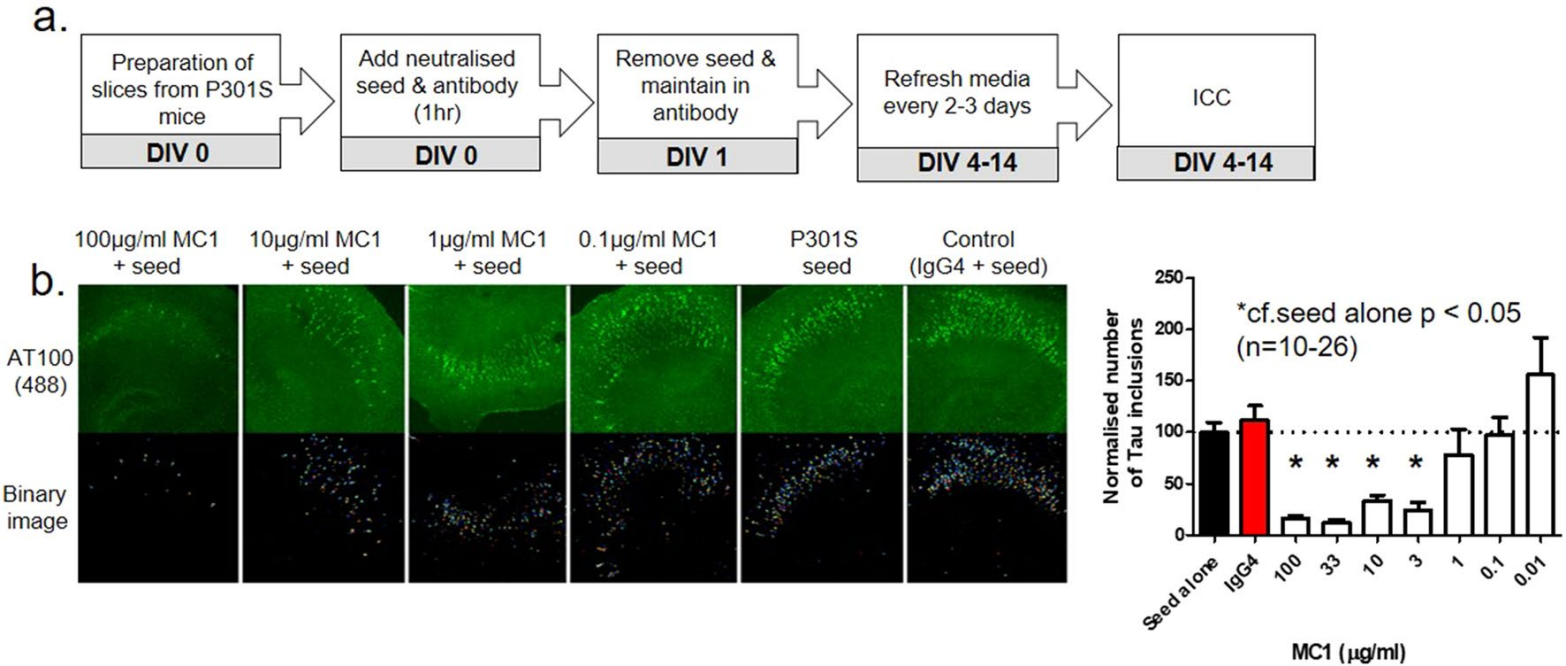
The MC1 antibody reduces AT100 + tau in a concentration-dependent manner. (**a**) Experimental paradigm of antibody neutralisation of P301S seed. (**b**) The MC1 antibody had the ability to reduce AT100 + tau inclusions in a concentration dependent manner as shown via immunocytochemistry (ICC). Statistics: Groupwise comparison, one-way ANOVA with Dunnett’s post hoc test; *, *P* < 0.05.

For this proof of principle experiment, we employed the conformation dependent anti-tau antibody MC1. Previously it has been shown that MC1 is effective in both in vivo and in vitro systems in neutralizing extracellular transmissible tau seeds^27^. We observed an antibody concentration-dependent decrease in AT100 positive tau inclusions when seed was neutralized with the MC1 antibody prior to inoculation of OHCs (Fig. 5; We verified this result via staining for another phosphorylated tau antibody, PG5; Supp Fig. 4). These studies demonstrate the potential of an antibody approach to halting the propagation of tau pathology and highlight how this multicellular 3D system could be a useful screening tool to identify and assess such antibodies.

## Discussion

The present study demonstrates how OHCs from P301S mice provide a useful ex vivo model to study tauopathy and the associated brain parenchyma. The slices show a robust and progressive pathology when inoculated with P301S seed extracted from terminally ill mice. The tau phenotype observed mirrors what has been reported in vivo*,* albeit in an accelerated time frame, which can be used to our advantage in the field of drug discovery.

We demonstrate that the P301S OHCs develop hyperphosphorylated insoluble tau in a dose and time dependent manner when seeded with brain homogenate from terminally ill P301S mice. The increase in insoluble phosphorylated tau over time suggests that tau may be propagating from cell-to-cell, which has been reported in vivo^6–8^. Another possibility is that different regions of the slice may have a delayed ability to form these tau inclusions upon seeding. The factors which potentially govern this are unknown. In the absence of seeding, P301S slices do not spontaneously form insoluble phosphorylated tau even after 3 months in culture. We suspect htau P301S mice OHC’s may need to be cultured for longer than three months to see insoluble phosphorylated tau^7,21^ or an additional stressor may be necessary to induce its formation which is lacking in the relatively benign culture conditions used. Time and relatively benign culture conditions may also explain the lack of cell death in our system—We did not observe any gross increase in cell death in the seeded slices versus unseeded slices (there was a trend towards an increase, but it was not significant). In the future it will be interesting to see if there are any subtle changes like decreases in dendritic spine density that precedes or succeeds the presence of tau pathology. Though it is worth noting that despite the fact that tau corelates quite well with cognitive decline in diseases such as Alzheimer’s, there is few convincing reports of tau induced cell death in ex vivo or in vitro model systems, suggesting other mechanisms/stressors may be required and accumulation of insoluble phosphorylated tau on its own is not sufficient to induce cell death.

The OHCs produced were functionally active, and some slices displayed spontaneous activity which could be blocked by a cocktail of NMDA/AMPA antagonists. It was beyond the scope of this present study to explore alterations of calcium dynamics and excitability in the seeded slices that display insoluble phosphorylated tau accumulations. Though we believe this would be an interesting area for future study, for example carrying out long term potentiation experiments looking at the effects of tau seeding, as other studies have suggested alterations here^25^. It would also be interesting to explore further the differences between slices displaying spontaneous activity and those did not in our experiments. All slices utilized were viable, showed similar staining via IHC for various cell lineage markers, and were capable of producing insoluble phosphorylated tau accumulations upon seeding. Whilst spontaneous synaptic activity has been reported before for OHCs^28^_,_ data on percentage of slices which display this, and why it may vary from slice to slice is relatively under reported.

The principle objective of establishing this ex vivo model was to aid in the development of novel therapeutics for tauopathies. To that end, we performed a proof of principle experiment to determine if our model could be used as a screening system to identify novel therapeutics. We utilized the conformational dependent anti-tau antibody, MC1, which has previously been shown to prevent tau seeding in in vitro and in vivo models^27^. We demonstrated that MC1 inhibited tau seeding in an antibody concentration dependent manner in our model, and was capable of completely preventing the formation of AT100 and PG5 positive tau. This correlates with what has been observed in in vivo studies^27^ and highlights the utility of our system in developing and testing such therapeutics. OHC’s behave and mimic what we see in vivo but have the advantage of being considerably more time and cost effective, suitable for low throughput screening paradigms in an industrial setting, as well as fulfilling the 3R’s of research.

## Methods

### Animals and slice preparation

All animal procedures were performed in accordance with the Animals (Scientific Procedures) Act 1986 and were approved by the Eli Lilly Animal Welfare Board. It was in compliance with the ARRIVE guidelines. Organotypic hippocampal slices were generated as described previously^29^. Briefly, 330 µM hippocampal slices were generated from P6-9 Tg P301S mice (hTau.P301S) and C57BL/6 J mice (both male and female pups). Slices were cultured in organotypic slice culture media^30^ via the interface method for up to 8 weeks at 37 °C, 5% CO_2_. Media was replaced 3 times/week.

### Preparation of brain extracts and seeding of slices

Brain extracts were prepared from end-stage (5–5.5 months) P301S tau mice and age-matched wild type controls (C57BL/6 J), as previously described^6,7^. In brief, mice were killed via dislocation of the neck and decapitation. Brains were rapidly removed and snap frozen on dry ice. Frozen whole brains from ten mice were combined and homogenized at 10% (w/v) in sterile phosphate-buffered saline (PBS), briefly sonicated (Branson 450, output 2, 5 × 0.9 s) and centrifuged at 3000 g at 4 °C for 5 min. The supernatant was assessed via AlphaScreen for total and phosphorylated tau levels and stored at − 80 °C until use. For the seeding of slices, slices were inoculated with brain homogenate in culture media immediately after preparation for 16 h at 37 °C, 5% CO_2_. Unless otherwise stated the seed was used at a final conc of 0.8 mg/ml total protein; 26.8 µg/ml total tau. After 16 h the inoculation media was replaced with regular culture media and slices cultured as normal.

### Antibodies

The following antibodies were kind gifts from Peter Davies (Albert Einstein College of Medicine, New York): total tau: DA9 (aa 102–140)^31^; TG5 (aa 220–240)^32^; phosphorylated tau: PG5 (pS409)^33^.

Phosphorylation-dependent anti-tau antibodies AT8 (pS202/pT205) and AT100 (pS212/pT214/pT217), were purchased from Thermo (Pierce).

### Biochemical analysis

At specified time points, slices were harvested and homogenized in sodium-phosphate buffer supplemented with protease (Roche) and phosphatase (Sigma-Aldrich) inhibitors. Fractionation of homogenates into insoluble and soluble proteins was achieved by a 1 h centrifugation at 100,000 g. Total and hyperphosphorylated tau were measured in the soluble fraction and the pelleted (insoluble) fraction by SDS-PAGE and Western blotting as described previously^16^. Samples were boiled in sample buffer containing 2-mercaptoethanol, loaded onto Novex 8% Bis–Tris gels (Life Technologies) and run at 150 V. Proteins were transferred onto nitrocellulose (GE Healthcare) in a semi-dry transfer tank, blocked with 5% fat-free milk (Marvel) and immunoblotted using AT8 and DA9. After secondary antibody incubation, bands were visualized using chemiluminescent substrate, and gels imaged and quantified using the ImageQuant LAS 4000 (GE Healthcare). For all immunoblots shown from OHCs, a minimum of 3 biological repeats on separate days were performed. For insoluble phosphorylated tau, a lot of material is needed as each individual slice yields only a small amount of material/protein and the fractionation process is not very efficient. Thus a minimum of 42 slices were combined per single lane of data displayed here in the immunoblots, principally to be sure we had enough material for the measurement of insoluble phosphorylated tau via fractionation. These slices came from a minimum of 6 different animals.

### Immunohistochemical analysis

Slices were fixed in 4% paraformaldehyde and 20% MeOH, permeabilized with 0.5% Triton-X-100 in PBS and blocked with 20% BSA in PBS. Slices were cut out of the insert and incubated with primary and secondary antibody as free-floating sections (on their membrane), cover-slipped and examined via confocal microscopy for tau inclusions (Olympus FluoView 1000; scanning speed 10 µs/pixel) or via the HCS Opera Phenix. For all IHC images shown, a minimum of 3 repeats were performed on different days. Each repeat consisted of imaging at least 6 slices per condition. All slices used per repeat came from a minimum of 3 different mice.

### Propidium iodide staining

At 14–28 days in vitro OHCs were treated with 1 mg/ml propidium iodide (PI) for 1 h before being assessed for viability with the BD pathway, a high content cell analyzer using a 4 × objective. Slices were continuously maintained in a sterile condition and at 37 °C when being taken out of the incubator to image. Post imaging slices were treated with 1 µm kainic acid and again with 1 mg/ml PI. Kainic acid is a potent kainite receptor agonist, and can act as a neurotoxin, killing neurons by overexcitation. This step was taken to confirm that the slices were indeed viable and contained live neurons. To ensure consistency and suitable comparability of experimental results, the same exposure settings were set during all experiments. For PI experiments, a minimum of 3 repeats were performed on different days. Each repeat consisted of imaging at least 6 slices per condition. All slices used per repeat came from a minimum of 3 different mice.

### Live-cell calcium imaging

Live-cell calcium imaging was largely performed as described previously for primary cells^34^. Briefly, Organotypic slices were submerged and loaded with 4 µM of a calcium-sensitive dye Fluo4-AM in the presence of 1% Pluronic acid (Invitrogen, Paisley, UK) diluted in HEPES buffered Tyrode’s solution (HBTS, Invitrogen, Paisley, UK) containing (mM): 135 NaCl, 5 KCl, 1.2 MgCl_2_, 2.5 CaCl_2_, 10 HEPES, 11 glucose, pH = 7.2. Slices were incubated for 1 h in the dark room, then washed two times with HBTS before recording.

Bipolar platinum-iridium electrodes (Science Products GmbH, Hofheim, Germany) were placed directly above the slices and used to deliver voltage pulses with defined characteristics (intensity and frequency) produced by a stimulus generator (NPI, Tamm, Germany). Dye-loaded cells were viewed using an inverted epifluorescence microscope (Axiovert 135TV, Zeiss, Cambridge, UK) as described previously^34^. Fluo-4 fluorescence was excited by a 480 ± 10 nm light source (Polychrome II, TILL-Photonics, Gräfelfing, Germany) and emission was captured by an iXon 897 EMCCD camera (Andor Technologies, Belfast, UK) after passage through a dichroic mirror (505LP nm) and a high pass barrier filter (515LP nm). A high acquisition frame rate of 10 frames/second was used during application of the EFS pulse train while slower rates were used between recordings (1 frame every 10 s). Digitized images were recorded and processed by using Imaging Workbench 5.0 software (INDEC Biosystems, Santa Clara, CA, USA). Delta F/F0 values were measured by calculating the ratio between the change in fluorescence signal intensity (delta F) and baseline fluorescence (F0).

### Antibody neutralization of seed

The filter-sterilized MC1 antibody was incubated at various concentrations with a single concentration of TgP301S seed (10 nM) and left to agitate for 1 h at room temperature on a benchtop shaker. The various concentrations were then added individually to a six well plate containing Millipore inserts that the slices were cultured on. The seed was removed after 24 h of culturing and replaced with only the concentration of antibody being tested. The media and antibody were replaced every 2–3 days up until the time of analysis via ICC.

### Statistical analysis

Quantitative data were analyzed using one-way ANOVA, followed by Dunnett’s post hoc tests for groupwise comparisons. GraphPad Prism 5 (version 5.04; GraphPad software Inc., CA, USA) was used to perform statistical tests and. Statistical significance was set at *P* < 0.05.

## Supplementary information

Supplementary Information.
