# Supplementary material for: Development of P301S tau seeded organotypic hippocampal slice cultures to study potential therapeutics
Source: Sci Rep. 2021 May 13;11:10309. doi: 10.1038/s41598-021-89230-3 (PMC8119691; doi:10.1038/s41598-021-89230-3)
Supplement: Supplementary file 1 — Supplementary Information. [file 41598_2021_89230_MOESM1_ESM.docx]

# **Supplementary figures**

**Development of P301S tau seeded organotypic hippocampal slice cultures to study potential therapeutics**S

.

James M. McCarthy*^1,2^, Jasmeet Virdee*^1^, Jessica Brown^1^, Daniel Ursu^1,4^, Zeshan Ahmed^1^, Annalisa Cavallini^1^, Hugh Nuthall^1#^**.**

*These authors contributed equally to this work

**^1^**Neuroscience, Eli Lilly and Company, Erl Wood Manor, Windlesham, Surrey, GU20 6PH, UK

^2^Current Address: Astex Pharmaceuticals, Milton Road, Cambridge, CB4 0QA.

^3^Current Address: Boehringer Ingelheim, Birkendorfer Straβe 65, Germany

^#^Corresponding author; [nuthall_hugh@lilly.com](mailto:nuthall_hugh@lilly.com)


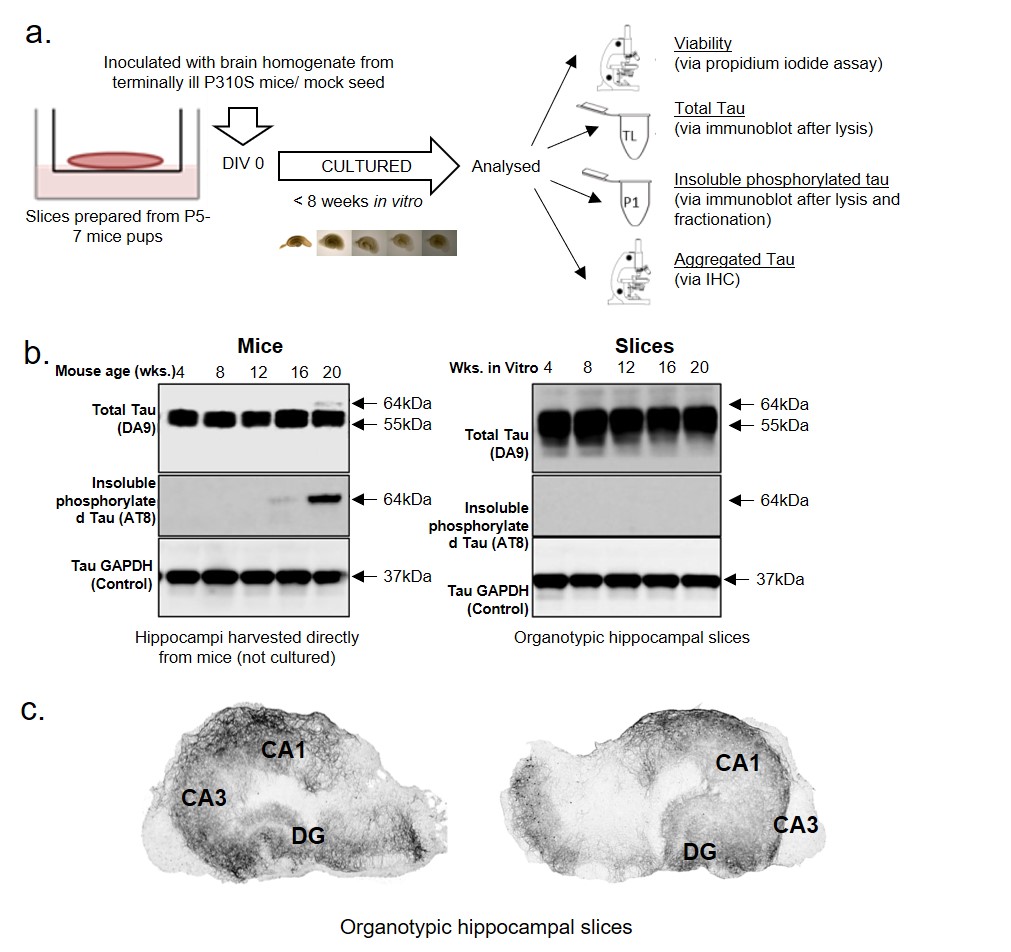


**Supp Figure 1. Human tau expression and phosphorylated tau production in P301S slices.**

1. A schematic depicting the timelines of seeding OHCs and the subsequent methods of analysis that were employed by this study. (b) Hippocampi from Tg P301S mice at various ages were harvested, lysed and fractionated via centrifugation at 100,000 g. Total lysate was examined via immunoblot for total tau levels (DA9) whilst the insoluble pellet from fractionation was examined for phosphorylated tau (AT8). OHCs produced from P7 Tg P301S mouse pups were cultured for up to 8 weeks before lysis and fractionation at 100,000 g. Total lysate was examined via immunoblot for total tau levels (DA9) whilst the insoluble pellet from fractionation was examined for phosphorylated tau. (c) OHCs cultured for three weeks showed uniform expression of total human tau (CP27) throughout the hippocampus


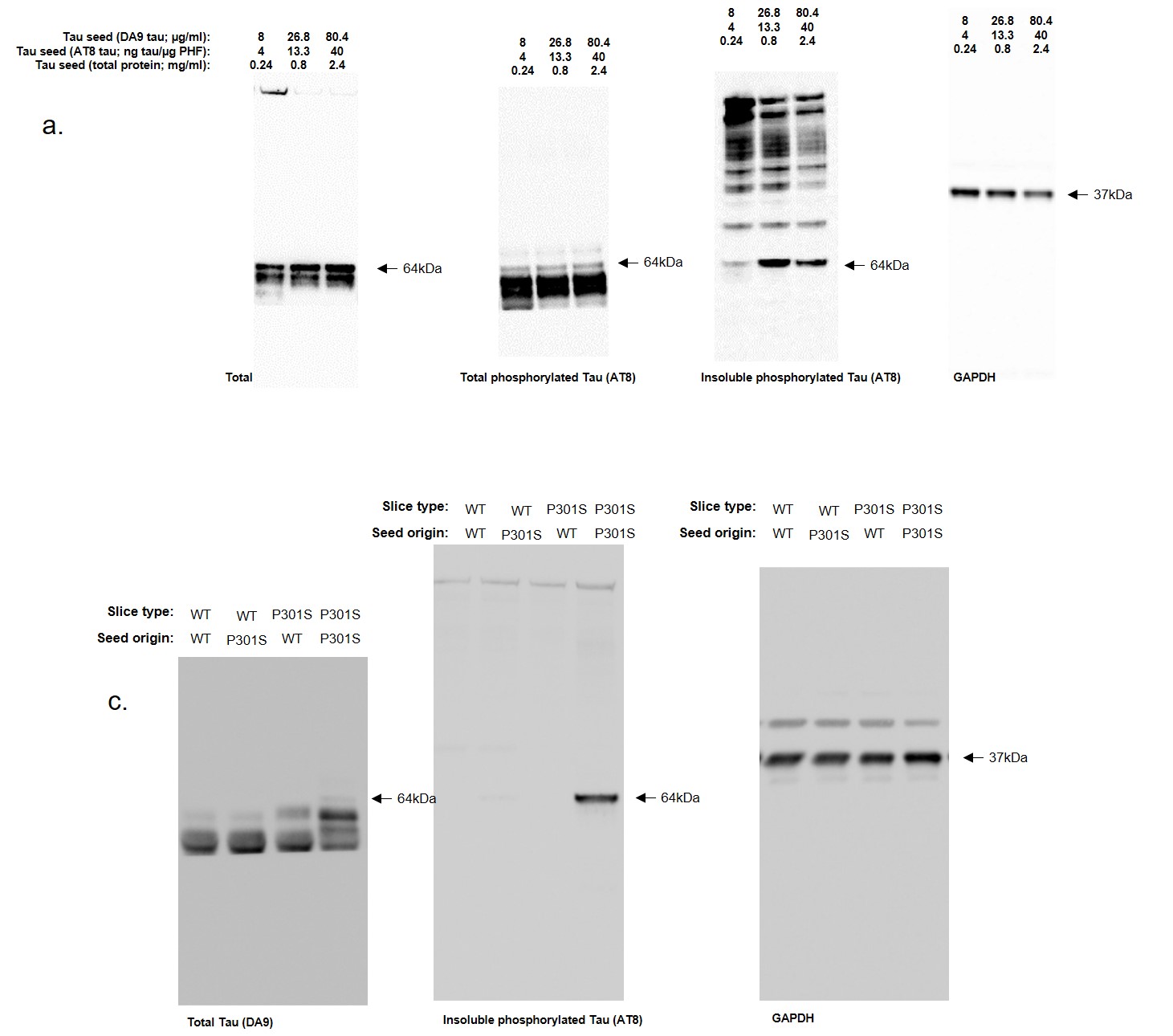


**Supp Figure 2. Original western blot images from Figure 3**


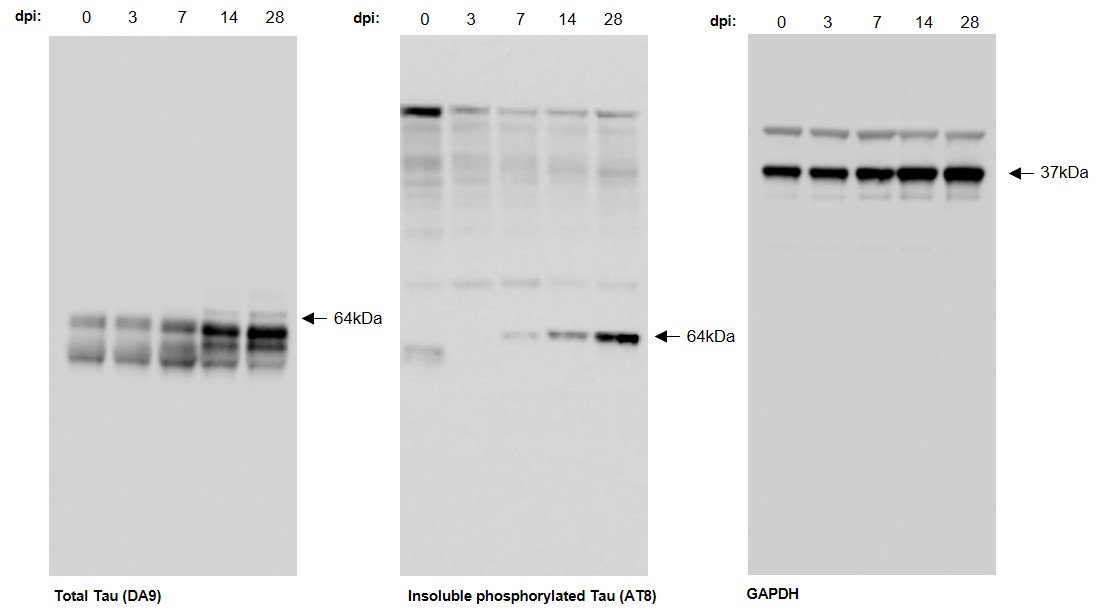


**Supp Figure 3. Original western blot images from Figure 4**


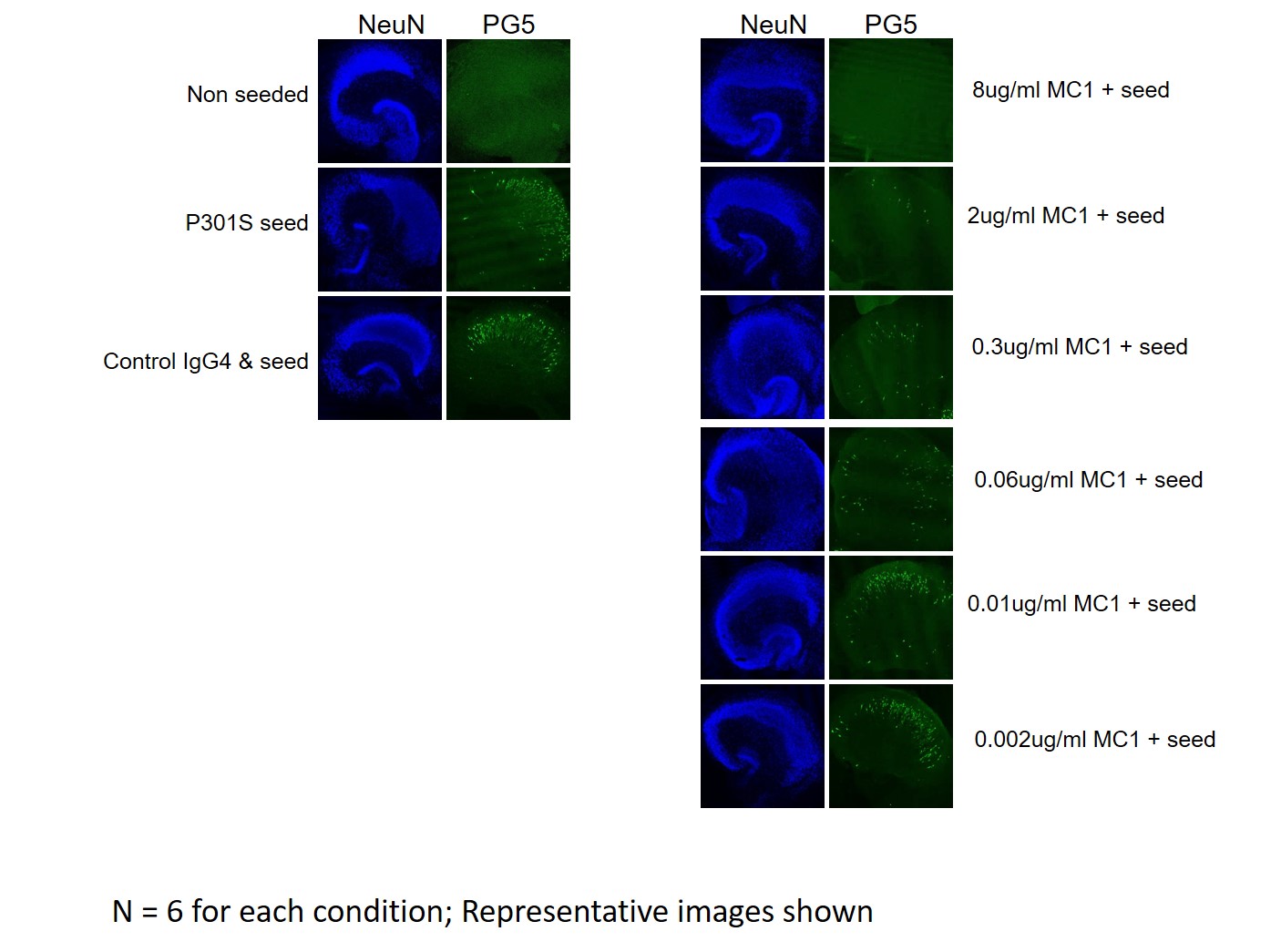


**Supp Figure 4. The MC1 antibody reduces PG5+ tau in a concentration responsive manner**

The MC1 antibody had the ability to reduce PG5+ tau inclusions in a concentration dependent manner as shown via immunohistochemistry (IHC).

**Supplementary Figure legends**

**Supp Figure 1. Human tau expression and phosphorylated tau production in P301S slices.**

(a) A schematic depicting the timelines of seeding OHCs and the subsequent methods of analysis that were employed by this study. (b) Hippocampi from Tg P301S mice at various ages were harvested, lysed and fractionated via centrifugation at 100,000 g. Total lysate was examined via immunoblot for total tau levels (DA9) whilst the insoluble pellet from fractionation was examined for phosphorylated tau (AT8). OHCs produced from P7 Tg P301S mouse pups were cultured for up to 8 weeks before lysis and fractionation at 100,000 g. Total lysate was examined via immunoblot for total tau levels (DA9) whilst the insoluble pellet from fractionation was examined for phosphorylated tau. (c) OHCs cultured for three weeks showed uniform expression of total human tau (CP27) throughout the hippocampus.

**Supp Figure 2. Original western blot images from Figure 3**

**Supp Figure 3. Original western blot images from Figure 4**

**Supp Figure 4. The MC1 antibody reduces PG5+ tau in a concentration responsive manner**

The MC1 antibody had the ability to reduce PG5+ tau inclusions in a concentration dependent manner as shown via immunohistochemistry (IHC).
